# Facilitation of Hippocampal Kindling and Exacerbation of Kindled Seizures by Intra-CA1 Injection of Quinine: A Possible Role of Cx36 Gap Junctions

**DOI:** 10.22045/ibj.2016.03

**Published:** 2016-11

**Authors:** Fatemeh Etemadi, Mohammad Sayyah, Hamid Gholami Pourbadie, Vahab Babapour

**Affiliations:** 1 Department of Physiology and Pharmacology, Pasteur Institute of Iran, Tehran, Iran; 2 Department of Physiology, Faculty of Veterinary Medicine, Tehran University, Tehran, Iran

**Keywords:** CA1, Connexin36, Gap junctions, Kindling, Quinine

## Abstract

**Background:**

GABAergic interneurons in the hippocampal CA1 area are mutually communicated by gap junctions (GJs) composed of connexin36 (Cx36). We examined the role of Cx36 in CA1 in manifestation of kindled seizures and hippocampal kindling in rats.

**Methods:**

Quinine, as the specific blocker of Cx36, was injected into CA1, and kindled seizures severity was examined 10 min afterward. Moreover, quinine was injected into CA1 once daily, and the rate of CA1 kindling was recorded.

**Results:**

Quinine 0.5 and 1 mM caused 2- and 3.5-fold increase in the duration of total seizure behavior and generalized the seizures. Primary and secondary afterdischarges (AD) were also significantly increased. Quinine 0.1 mM augmented the rate of kindling and the growth of secondary AD.

**Conclusion:**

Cx36 GJs in CA1 are the main components of hippocampal inhibitory circuit. Any interruption in this path by pathologic or physical damages can trigger hippocampal hyperexcitability and facilitate epileptogenesis. xx

## INTRODUCTION

Epilepsy is one of the most common neurological disorders affecting 1% of the population. Understanding the mechanisms underlying epileptogenesis helps in designing effective medications for fundamental, not symptomatic, therapy of epilepsy[1].

For years, chemical synaptic transmission has been considered as the main mechanism involved in aberrant neuronal synchrony in seizures. Later, the role of gap junctions (GJs) was elucidated[2,3]. Every six connexin (Cx) proteins aggregate at the cell membrane form a hemichannel called connexon, which associates with another connexon on a neighboring cell to form a mature GJ. GJs allow intercellular movement of ions, metabolites and second messengers. Among Cxs, Cx36 is exclusively expressed in neurons, especially interneurons[4]. GJs contribute to ictogenesis via facilitating the rapid propagation of electrical activity between neurons and increasing synchronous activity[2]. GJs are also important for seizure initiation[5]. Rhythmic hippocampal oscillations induced by 4-AP are different between wild-type and Cx36 KO mice[6]. GJ blockers inhibit neural population activity[3,7,8]. Conversely, Cx36 GJs have an anticonvulsant role in excitotoxic conditions so that Cx36 knockout mice show enhanced sensitivity to pentylenetetrazole-induced seizures[9]. Cx36 is considered as a genetic marker of the juvenile myoclonic epilepsy[10], and blockade of Cx36 GJs in CA1 accelerates amygdala kindling[11].

Hippocampus has a pivotal role in epileptogenesis[1]. There is a network of GJs between different cell types within the hippocampus[3,4]. Inhibitory GABAergic interneurons comprise substantial fraction of hippo-campal cells and express Cx36. GJs in CA1 neurons may play important role in hyper-synchronization of epileptiform activity[8,12]. Alteration in the expression of Cxs during seizure activity is proposed as a possible mechanism underlying neuronal synchronization[13-15]. In this line, Cx36 expression is elevated during epileptogenesis provoked by amygdala kindling[13]. Yet there is little known about the role of hippocampal Cx36 on epileptogenesis during hippocampal kindling. We evaluated the impact of Cx36 GJs in CA1 on development of CA1 kindling as well as CA1-kindled seizures severity.

## MATERIALS AND METHODS

### Animals

Adult male Wistar rats (280-350 g, Pasteur Institute of Iran, Tehran) were housed in cages with free access to food and water and maintained at 23±1°C with a 12 h light/dark cycle. The study was approved by the Ethics Committee of Pasteur Institute of Iran and conforms to the European Communities Council Directive of 24 November 1986 (86/609/EEC).

### Rapid kindling procedure

Rats were anesthetized with intraperitoneal (i.p.) injection of 60 mg/kg ketamine and 10 mg/kg xylazine. Bipolar stimulating and monopolar recording stainless-steel Teflon-coated electrodes, which was attached longitudinally to a cannula, were stereotaxically implanted in the dorsal CA1, based on the previously described method[11]. The kindling procedure was started one week afterward. Rats were stimulated at the afterdischarge (AD) threshold (100-300 μA) by 12 repetitive daily stimulations with a 30-min interval between each stimulation, until three consecutive stage 5 (generalized) seizures were elicited[11]. Quinine was dissolved in artificial cerebrospinal fluid (ACSF) composition (in mM): 124 NaCl, 2 CaCl_2_, 4.4 KCl, 2 MgCl_2_, 1.25 KH_2_PO_4_, 25 NaHCO_3_ and 10D glucose, and infused (1 μl/5min) into dorsal CA1. Seizure parameters, including primary AD duration (ADD), secondary ADD, stage 5 duration, seizure duration and behavioral seizure stages were recorded.

### Intra-CA1 injection of quinine to kindled rats

Four groups of seven kindled rats were treated by 1 μl ACSF (control) or quinine 0.1, 0.5 and 1 mM into CA1. The animals were stimulated by threshold current 10 min later, and seizure parameters were recorded.

### Intra-CA1 injection of quinine during kindling

After determination of AD threshold, the animals were divided into three groups of six rats in each. The rats received quinine (0.03 and 0.1 mM/rat, intra-CA1) or ACSF (1 µl/rat, intra-CA1) once daily 10 min after the completion of daily electrical stimulations. The procedure was continued until the animals became fully kindled. Seizure parameters were recorded after each stimulation.

### Histology

At the end of the experiments, the brains were removed, processed, cut into 50-µm thick slices and stained by Toluidine blue. Cannula and electrode positions and any lesion were qualitatively analyzed using stereoscopic and light microscopes (Zeiss, Germany). The data of the animals with false placement of cannula and electrode were excluded from the results.

### Data analysis

Data were presented as mean±SEM. The kindled seizure parameters, including primary and secondary ADD, stage 5 duration, seizure duration and the number of stimulations required for the acquisition of generalized seizures were analyzed by one-way ANOVA, followed by Bonferroni’s post-test. Primary and secondary ADD during kindling development were analyzed by two-way ANOVA with Bonferroni’s post-test. In all experiments, *P*<0.05 was considered statistically significant.

## RESULTS

### Histology

Histological evaluation showed a correct position of electrodes and cannula in the CA1 area. No dramatic morphological changes and neuronal loss were observed in the CA1 following consecutive microinjections of quinine.

### Excitatory effect of quinine in kindled rats

**Fig. 1:**
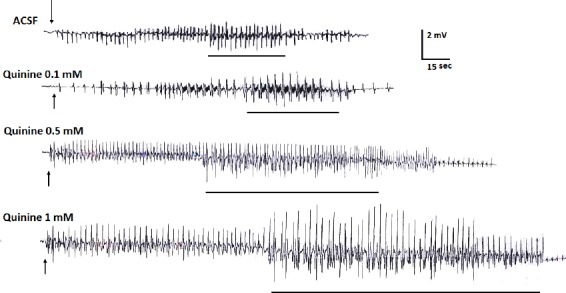
Primary afterdischarges (ADs) recorded from dorsal CA1 of kindled rats. Quinine 0.5 and 1 mM significantly increased the severity and the duration of primary and secondary ADs. Lines under traces show the period of generalized seizures in a whole record of primary ADs. Arrows represent the time point of stimulation. ACSF, artificial cerebrospinal fluid.

**Fig. 2:**
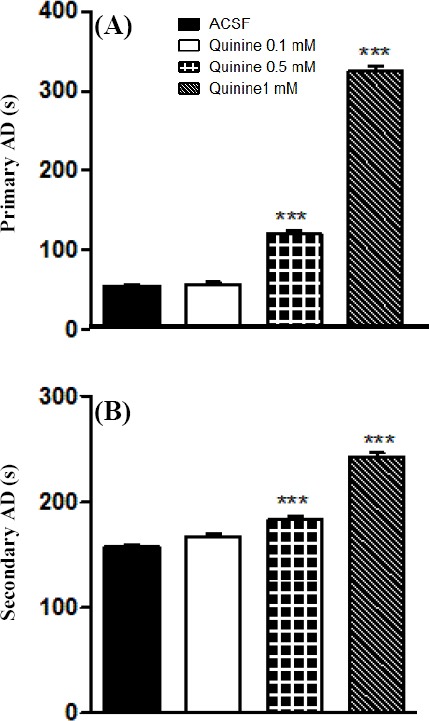
Effects of intra-CA1 microinjection of quinine on duration of primary and secondary afterdischarge (AD) in the kindled rats. Quinine 0.5 and 1 mM significantly increased primary (A) and secondary (B) AD duration. ***P<0.001 compared to ACSF group. Data represent mean±S.E.M (n=7). ACSF, artificial cerebrospinal fluid

**Fig. 3:**
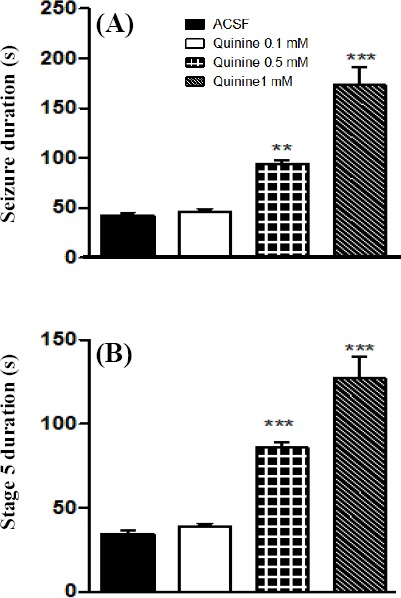
Effect of intra-CA1 injection of quinine on kindled seizures. (A) Total seizure duration was significantly increased by quinine 0.5 and 1 mM. (B) Duration of generalized seizures was prolonged by quinine 0.5 and 1 mM. **P<0.01 and ***:*P*<0.001 compared to ACSF group. Data represent the mean±SEM (n=7). ACSF, artificial cerebrospinal fluid

Quinine 0.5 and 1 mM significantly increased the severity and the duration of primary and secondary AD (Figs. 1 and 2). Duration of stage 5 seizures as well as total seizure behavior was also significantly increased by quinine 0.5 and 1 mM (Fig. 3).

### Effect of quinine on development of hippocampal kindling

**Fig. 4:**
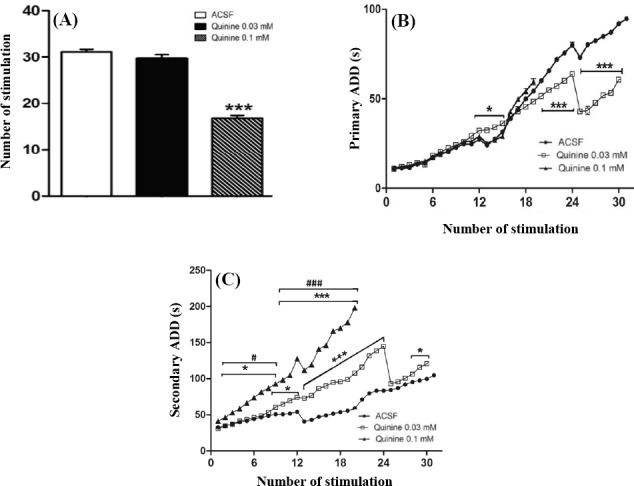
Effect of intra-CA1 microinjection of quinine on the kindling rate. (A) Daily microinjection of quinine 0.1 mM significantly decreased the number of stimuli required to elicit generalized seizures; (B) Quinine 0.03 mM significantly inhibited the growth of primary AD from the 20^th^ stimulation onward, and then a significant drop was observed in the trend at 24^th^ stimulation; (C) Both doses of quinine significantly increased the duration of secondary AD. *P<0.05 and ***P<0.001 compared to ACSF group. #*P*<0.05 and ###*P*<0.001 compared to quinine 0.03 mM group. A significant fall-down in the growing trend of secondary AD is prominent in quinine 0.03 mM group at 24^th^ stimulation. Data represent mean±SEM. Six rats were used in each experimental group. In ACSF (control) group, all six rats were kindled within 31 stimuli. In quinine 0.1 mM group, all 6 rats need about 19 stimuli to become kindled. In quinine 0.03 mM group, 5 rats need 30 stimuli and 1 rat required 36 stimuli to become kindled. The data from 32 to 36 stimuli for quinine 0.03 mM group are not shown in parts B and C. ACSF, artificial cerebrospinal fluid

Daily intra-CA1 injection of quinine 0.1 mM significantly decreased (*P*<0.001) the number of stimulations required to achieve generalized seizures (Fig. 4A). Quinine 0.1 mM had no significant effect on the growth of primary AD. The dose of 0.03 mM significantly increased the development of primary AD from the 12^th^ till 15^th^ stimulation (*P*<0.05, Fig. 4B). The growth of primary AD was then inhibited from 20^th^ stimulation onward (Fig. 4B). A significant fall-down in the growth of primary AD was observed from 24^th^ stimulation and then continued. There was a significant interaction between treatment and the number of stimuli [F(36,228)=10.94; *P*<0.001] and between treatment and the growth of primary AD [F(2,228)=21.45; *P*<0.001]. Quinine 0.03 and 0.1 mM significantly increased the development of secondary AD during kindling (Fig. 4C). A significant interaction between treatment and the number of stimuli [F(38,228)=244; *P*<0.001] and between treatment and the growth of secondary AD [F(2,228)=1582; *P*<0.001] were found. Bonferroni’s post test revealed that increase in secondary AD in quinine 0.1 mM group was significant from the second stimulation onward. In quinine 0.03 mM group, the raise was prominent from 9^th^ till 24^th^ stimulation. Then a significant drop in growing of secondary AD happened at 24^th^ stimulation since from this time point, the trend was close to control (ACSF) and then again a significant raise in secondary AD emerged from 27^th^ stimulation and continued (Fig. 4C).

## DISCUSSION

We found that intra-CA1 injection of quinine significantly increases the duration of ADs, generalized seizures as well as the total seizure behavior in kindled rat. Moreover, quinine accelerates the rate of hippocampal kindling. It is unlikely that any direct excitotoxicity underlies the observed effects of quinine, as no histopathological alteration was seen in the quinine-treated brain slices. In accord, it has been reported that quinine has no excitotoxic activity even protects neural cells[16].

Blocking Cx36 GJs in CA1 accelerates amygdala kindling[11]. Compared to amygdala kindling, different sets of networks are involved in hippocampal kindling. Hippocampal inhibitory network is essential for shaping the output signal. The hallmark of hippocampus is fast neuronal oscillations emerging from a random network of interconnected GABAergic fast-spiking interneurons[17]. There are three types of parvalbumin-expressing (PV+) interneurons in the hippocampal formation, i.e., PV+ basket cells, bistratified cells, and axo-axonic cells, which are involved in generation of gamma rhythm and ripples. All of these cells present electrophysiological characteristics of fast-spiking cells and establish connections by GJs containing Cx36 within their groups[18]. GJs are involved in the fast oscillations preceding the onset of seizures discharges in the hippocampus[19,20]. In Cornu Ammonis, inhibitory GABAergic interneurons develop diverse dendrodendritic connections with other interneurons, and pyramidal cells result in synchronized oscillations promoting feedback and feed forward inhibition[21,22]. Electrical coupling between GABAergic interneurons in this region is mediated by Cx36 GJs[22]. GJ between inhibitory interneurons protects neurons against excitotoxicity in the pathologic conditions such as seizures[23]. Blocking Cx36 GJ in CA1 might attenuate the inhibitory circuit and provokes hyperexcitability of principal neurons and propagation of aberrant discharge to neighboring areas. Accordingly, Cx36 knockout mice show a reduced GABAergic tone, a less inhibitory control of excitatory runaway activity, and predisposing to generalized seizures[24,25] and decreased threshold of pentylenetetrazole-induced generalized seizures[9]. Cx36 is also frequently expressed in cerebral cortex in a subclass of inhibitory interneurons named parvalbumin-containing basket cells[26-28]. Interestingly, quinine strengthens the frequency and the amplitude of seizure-like activity in rat cerebral cortex[28]. However, there are some controversial reports that blocking Cx36 GJs attenuates seizures[8, 29-31]. Quinine decreases duration of seizures elicited by 4-AP[29], pentylenetetrazole[30] and penicillin[31]. Furthermore, the amplitude and the frequency of epileptic discharges are dampened following entorhinal microinjection of quinine in seizing rats[8]. This discrepancy might arise from using different models of seizures and hence the involvement of different seizure mechanisms without involvement of inhibitory interneurons[8].

Quinine increased the duration of both primary and secondary AD in kindled rats in a dose-dependent trend. Secondary AD in hippocampus is a robust phenomenon and believed to be a reflection of reverberating AD activity from regions to which the primary AD spreads e.g., entorhinal cortex[32]. Secondary AD is largely generated in hippocampus by perforant path at synapses of dentate gyrus with variable spreading to CA1[32]. Thus, lengthening of primary AD caused more spread of discharges to extra CA1 regions, and this in turn results in more return of inputs to CA1 and consequent increase in duration of secondary AD.

Chronic intra-CA1 injection of quinine during kindling accelerates the rate of hippocampal kindling and increases the growth of secondary AD. However, the growth of primary AD was not affected and even conversely slowed down by the low dose of quinine. There might be some explanations for paradoxical effect of quinine on the growth of primary AD at the low dose. While primary AD in hippocampus is originated from CA1, secondary AD is largely generated by perforant path [32]. It is likely that diverse mechanisms govern the growth of primary and secondary AD in hippocampus. Suppression of primary AD development by quinine 0.03 mM might be mediated by mechanisms other than the blockade of Cx36 GJs. Quinine blocks not only Cx36 GJ at 100 µM but also Cx45, IP_3_-induced Ca^2+^ release, Cx50, sodium currents, voltage-dependent K^+^ channels, ATP-sensitive K^+^ channels and nicotinic receptors at the different ranges of concentrations[33]. It is likely that opposite effects of quinine on the growth of hippocampal AD at the low dose (0.03 mM) is related to act on the other targets than Cx36. In conclusion, inhibition of Cx36 GJs in CA1 accelerates CA1 kindling and intensifies kindled seizures in rats. Hippocampal Cx36 GJs seem to be a crucial element in development of epilepsy and seizure severity.
